# Released Myeloperoxidase Attenuates Neutrophil Migration and Accumulation in Inflamed Tissue

**DOI:** 10.3389/fimmu.2021.654259

**Published:** 2021-04-20

**Authors:** Jacob F. Rehring, Triet M. Bui, Carlos Samuel Galán-Enríquez, Jessica M. Urbanczyk, Xingsheng Ren, Hannah L. Wiesolek, David P. Sullivan, Ronen Sumagin

**Affiliations:** Department of Pathology, Northwestern University Feinberg School of Medicine, Chicago, IL, United States

**Keywords:** neutrophils, adhesion, migration, inflammation, imaging, myeloperoxidase (MPO)

## Abstract

Neutrophil (PMN) recruitment to sites of insult is critical for host defense, however excessive PMN activity and tissue accumulation can lead to exacerbated inflammation and injury. Myeloperoxidase (MPO) is a PMN azurophilic granule enzyme, which together with H_2_O_2_, forms a powerful antimicrobial system designed to kill ingested bacteria. Intriguingly, in addition to intracellular killing of invading microorganisms and extracellular tissue damage due generation of ROS, soluble MPO has been directly implicated in modulating cellular responses and tissue homeostasis. In the current work, we used several models of inflammation, murine and human PMNs and state-of-the-art intravital microscopy to examine the effect of MPO on PMN migration and tissue accumulation. We found that in the absence of functional MPO (MPO knockout, KO mice) inflammatory PMN tissue accumulation was significantly enhanced. We determined that the elevated numbers of PMNs in MPO knockout mice was not due to enhanced viability, but due to increased migratory ability. Acute PMN migration in models of zymosan-induced peritonitis or ligated intestinal loops induced by intraluminal administration of PMN-chemokine CXCL1 was increased over 2-fold in MPO KO compared to wild type (WT) mice. Using real-time intravital imaging of inflamed mouse cremaster muscle and *ex vivo* PMN co-culture with inflamed endothelial cells (ECs) we demonstrate that elevated migration of MPO KO mice was due to enhanced adhesive interactions. In contrast, addition of soluble recombinant MPO both *in vivo* and *ex vivo* diminished PMN adhesion and migration. Although MPO has been previously suggested to bind CD11b, we found no significant difference in CD11b expression in either resting or activated PMNs and further showed that the MPO binding to the PMN surface is not specific to CD11b. As such, our data identify MPO as a novel regulator of PMN trafficking in inflammation.

## Introduction

Inflammatory responses feature rapid mobilization of neutrophils (PMNs) towards the insult sites (1). While PMNs fulfill essential roles in combating invading pathogens, providing the first line of defense to the host, they can also promote exacerbated inflammation resulting in tissue damage. PMN-associated tissue damage occurs mainly due to the release of various proteinases, including matrix metalloproteinases (MMPs), Elastase and Myeloperoxidase (MPO) into the surrounding tissue (2, 3)

MPO is the most abundant protein expressed by PMNs and perhaps one with the most damaging/cytotoxic potential to living cells. Together with H_2_O_2_, MPO forms a powerful antimicrobial system designed to kill ingested bacteria. MPO is stored in the azurophilic/primary granules and is released into the phagosomes, where it catalyzes the conversion of H_2_O_2_ to hypochlorous acid (HOCl) (4) Although it is safeguarded in the azurophilic/primary granules, which require stronger stimulation and are the last to be released during the hierarchical release of PMN granules (5), plenty of evidence suggests spilling of free MPO into the surrounding tissue during inflammation (6). As such, elevated plasma and tissue levels of MPO were detected in many inflammatory disorders, where the presence of MPO or its oxidative derivatives have been assigned pro-inflammatory or in some cases protective functions [summarized in (7)].

Importantly, in addition to the intracellular killing of invading microorganisms and extracellular generation of ROS, emerging evidence indicates that MPO can directly modulate cellular function and immune responses, making it both an attractive disease biomarker and a therapeutic target. Several new functions were identified for free MPO, which include modulation of endothelial and epithelial responses (8, 9) as well as macrophage and dendritic cell activation (10, 11). However, MPO paracrine modulation of PMN activity particularly stands out. Free MPO has been shown to bind CD11b at the PMN surface and *via* these interactions, trigger MAPK- and NFκB-dependent ROS production and degranulation by PMNs (12) or improve phagocytosis *via* the induction of FAK/ERK signaling (13). MPO has also been shown to provide adhesive support for PMNs (14) and modulate both Ca^2+^ levels and cytoskeleton organization (15), suggesting its potential role in PMN trafficking.

Since for many inflammatory disorders, resolution of inflammation is an important clinical endpoint and PMNs can both exacerbate inflammation and initiate reparative responses, understanding mechanisms that govern the trafficking and recruitment of these cells in tissue are of ongoing clinical interest. To mediate either the protective or pro-inflammatory action, PMNs must first exit the circulation by crossing the endothelial layer. Transendothelial migration (TEM) is a highly regulated process that is initiated with the capture of free-flowing PMNs at the vessel wall, followed by slow rolling and adhesion to ECs and terminated with migration across the EC monolayer [reviewed in (16)]. The PMN recruitment cascade is well-studied and the signaling and ligands mediating this process are well-recognized, however specific approaches to target PMN migration are still under investigation.

Given the implication of excessive PMN infiltration and activity in tissues in inflammatory disorders and the newly emerging evidence of MPO in modulating the PMN function, in the current work we thought to examine the impact of MPO on PMN trafficking and recruitment in inflammation. We utilized two murine models of sterile inflammation and murine and human PMNs to definitively show that in the absence of MPO the adhesive and migratory ability of PMNs is enhanced, resulting in elevated tissue accumulation. Furthermore, free MPO was found to significantly suppress PMN migration, independently of its binding to CD11b. As such, although additional studies are needed to define the specifics of MPO bindings to the PMN surface, our findings identify a new and physiologically relevant role of MPO in PMN trafficking in inflammation.

## Materials and Methods

### Animals

C57BL6J mice, MPO knockout mice (B6.129X1-*Mpo^tm1Lus^*/J) (Jackson Laboratories) and LysM-eGFP reporter mice (Lyz2tm1.1Graf, gift from Dr. Perlman, Northwestern University) ages 8–16 weeks, were maintained under specific pathogen-free conditions at Northwestern University, Feinberg School of Medicine animal facilities. At the end of all experimental procedures, animals were euthanized *via* rapid cervical dislocation. All experimental protocols were approved by the Institutional Animal Care and Use Committee.

### Cells

Mouse microvascular brain endothelial cell (Bend-3) were grown in Dulbecco’s modified Eagle’s medium (DMEM) supplemented with 10% fetal bovine serum (FBS), 1% L-glutamine, and 1% penicillin-streptomycin. Human umbilical endothelial cells (HUVECs) were grown in human endothelial SFM supplemented with 1% L-glutamine, endothelial cell growth supplement (0.0375 mg/mL), 10% FBS, 1% penicillin-streptomycin, and 1% non-essential amino acids. *Human PMNs* were isolated from blood obtained from healthy volunteers by density gradient centrifugation (17, 18) and handled according to protocols for the protection of human subjects, as approved by the Northwestern University Institutional Review Board. PMNs were used for experiments within 2 hours of isolation. *Murine Bone Marrow PMNs* were isolated from bone marrow and enriched to ~85-90% purity using Histopaque gradients (1077 and 1119, Sigma) as previously described (19).

### Antibodies and Reagents

RPMI 1640, Dulbecco’s modified Eagle’s medium (DMEM) growth media, human endothelial SFM, endothelial cell growth supplement, L-glutamine, penicillin, streptomycin, nonessential amino acids, zymosan, collagen, Hanks balanced salt solution with/without Ca^2+^ and Mg^2+^ (HBSS+, HBSS-), Annexin V FITC, antiS100A8, and Propidium Iodide (PI) were obtained from Fisher Scientific (Waltham, MA). N-formyl-l-methionyl-leucyl-l-phenylalanine (fMLF) and Phorbol 12-myristate 13-acetate (PMA) were acquired from Sigma-Aldrich (St. Louis, MO). Human/murine interferon gamma (IFNγ) and tumor necrosis factor alpha (TNFα) and murine PMN chemoattractant CXCL1 (KC) were obtained from PeproTech (Rocky Hill, NJ). Bovine Serum Albumin (BSA) was obtained from Gemini Bio-Products (West Sacramento, CA). CD11b blocking antibody (clone M1/70), Pacific Blue™ anti-mouse CD45 antibody (clone 30-F11), APC anti-mouse CD144 (VE-cadherin, clone BV13), APC anti-mouse CXCR2 (clone SA044G4), PerCP cy5.5, anti-mouse CXCR4 (clone L276F12) and Alexa Flour 488 anti-His Tag (Clone J099B12) were from BioLegend (San Diego, CA). The rat IgG2b isotype was from BioXCell (Lebanon, NH). Recombinant mouse/human MPO protein, recombinant mouse interleukin 1-beta (IL-1β) and anti-h/m cleaved caspase Ab (Asp-135, clone 269518) were purchased from R&D systems (Minneapolis, MN). Anti-mouse PECAM-1 was purchased from Merck KGaA (Darmstadt, Germany) and labeled with DyLight 650 Fluorochrome purchased from Thermo Fisher Scientific (Waltham, MA). PE anti-CD11a (clone 2D7) and anti-Ly6G Abs (clone 1A8) were from BD Biosciences (San Jose, CA, USA). Anti-CD11b antibodies and rat monoclonal E-cadherin (DECMA-1) were from Abcam Inc. (Cambridge, MA). Horseradish peroxidase conjugated anti-mouse/rabbit secondary Ab was purchased from Jackson ImmunoResearch (West Grove, PA). Polyclonal anti-mouse p44/42 MAPK (ERK 1/2) and anti-mouse phospho-p44/42 MAPK (pERK 1/2; clone D13.14.4E) Abs were from Cell Signaling (Danvers, MA). Polymorphprep was purchased from Axis-Shield (Alere Technologies, Oslo, Norway). Fetal bovine serum (FBS) was obtained from Atlanta Biologicals (Atlanta, GA). Ketamine and xylazine were obtained from Henry Schein (Dublin, OH) and Akorn (Decatur, IL), respectively.

### Immunofluorescence Staining

*For intestines*, inflamed loops were OCT-frozen and 10µm-thick sections were blocked with 5% bovine serum albumin (BSA) in PBS, and incubation with the relevant primary antibody (10µg/ml, overnight, 4°C) followed by secondary antibodies (2hr, RT). *For cremaster muscle*, whole mount tissue was lightly permeabilized and blocked using PBS containing 0.3% Triton X-100, 2% BSA, and 1% goat serum overnight at 4°C. The samples were then incubated with primary antibodies (CD31 and S100A8) overnight at 4°C followed by secondary antibodies in permeabilization/blocking buffer (4h, RT). *For isolated PMNs*, BM-derived PMNs (3×10^5^ cells per condition) were mounted on slides, fixed with 4% paraformaldehyde followed by permeabilization using 1% Triton X-100 and stained with the relevant primary antibody (10µg/ml, overnight, 4°C) followed by secondary antibodies (2hr, RT). All tissues were mounted on slides using FluorSave (EMD Millipore) for imaging. Imaging was performed using UltraVIEW VoX imaging system built on an Olympus BX-51WI Fixed Stage illuminator and equipped with a Yokogawa CSU-X1-A1 spinning disk, a Hamamatsu EMCCD C9100-50 camera, and a Modular Laser System with solid state diode lasers with DPPS modules for 488, 561, and 640 nm and the appropriate filters (all assembled by Perkin Elmer, Naperville, IL).

### Intravital Imaging

For imaging of the cremaster muscle, which is a muscle surrounding the testis, male mice were used. Inflammation of the cremaster muscle was induced by intrascrotal injection of IL-1β (50ng, 4hr). When specified, murine recombinant MPO or denatured MPO protein (10 min, 95°C) was also administered immediately following stimulation (30µg/ml, iv). The ketamine and xylazine mixture (100mg/kg and 5mg/kg body weight, respectively) was used as initial anesthetic and to subsequently maintain appropriate anesthesia levels throughout the imaging procedures. The cremaster muscle was surgically exposed and prepared for imaging as previously described (20). Animal body temperature during imaging was maintained at 37°C and the tissue was continuously hydrated by superfusion of warmed perfusion buffer consisting of Tyrode’s salts (Sigma Aldrich) and 1 g/l sodium bicarbonate (pH 7.4; Thermo Fisher Scientific, Waltham, MA). *Imaging* was performed using Olympus BX-51WI Fixed Stage illuminator and equipped with a Yokogawa CSU-X1-A1 spinning disk, a Hamamatsu EMCCD C9100-50 camera, and a Modular Laser System with solid state diode lasers with DPPS modules for 488, 561, and 640 nm and the appropriate filters (all assembled by Perkin Elmer, Naperville, IL) as previously described (21). Synchronization was managed by a Prosync 2 Synchronization Controller. Z-axis movement and objective positioning was controlled by Piezoelectric MIPOS100 System (Piezoystem Jena, Germany). Images were collected using a 20x water-immersion objective (1.00 numerical aperture). Volocity^®^ software (Perkin Elmer) was used to drive the microscopy and acquire images, which were then analyzed using ImageJ.

#### Analyses of PMN-EC Interactions

For analyses of cell fluxes, PMN rolling and adhesion, 30sec recording of random fields containing a 30- to 50-μm postcapillary venule with steady flow were made using brightfield illumination and acquisition rate of 15 frames per second. Cell flux was defined as the number of cells that were visualized in the field of view, per 30 sec acquisition time. Rolling cells were defined as cells that have remained in continued contact with the vessel wall for greater than 5 seconds. Adherent cells were defined as cells that remained attached to the vessel wall for >20 sec. For analyses of transmigrated/tissue leukocytes, cells were quantified along 100μm vessel segments and within 50μm of the vessel of interest. Of note, it has been previously established that ~90% of all transmigrated leukocytes in IL-1β-stimulated cremaster muscles (4-5r hour stimulation) are neutrophils (22).

### Sterile Intestinal Inflammation Model

Inflammation in the intestine was induced by intraperitoneal administration of TNFα and IFNγ (500ng each, 24hr) as has been previously described (23, 24). TNFα and IFNγ are the most dominant cytokines in intestinal inflammation and injury and such stimulation truthfully simulates human pathology as seen in Inflammatory Bowel Diseases.

### Ligated Ileal Loop Model

Animals were anesthetized by ketamine and xylazine mixture (100 and 5 mg/kg, ip., respectively). A midline abdominal incision was made, and a 2-cm loop of small intestine was exteriorized and clipped at proximal and distal ends. After luminal administration of PMN chemoattractant CXCL1 (KC, 1μM in 200μl HBSS+), excised loops were reinserted into the peritoneal cavity (21). After 1-2 hours incubation, luminal lavage samples were taken and analyzed by flow cytometry. PMNs were defined and gated as CD45^+^/CD11b^+^/Ly6G^+^ cells.

### Zymosan-Induced Peritonitis Model

Zymosan injection (2µg/ml in 500μl PBS, ip.) was used to induce peritoneal inflammation and PMN recruitment into the peritoneal cavity. Four hours following Zymosan administration, peritoneal content was lavaged using 3 mL of PBS supplemented with 10% FBS and the number of migrated PMNs was quantified using flow cytometry. PMNs were defined and gated as CD45^+^/CD11b^+^/Ly6G^+^ cells.

### Flow Cytometry

Single cell suspensions following the relevant treatment were analyzed using a BD LSR Fortessa X-20 (Becton Dickinson, Franklin Lakes, NJ) instrument and FlowJo 10.7 software (Becton Dickinson).

### Transwell Chemotaxis Assay

Transwells (3.0µm pore size, CoStar Group, Washington DC) were coated overnight with 1µg/µl collagen in 0.2% acetic acid buffer. Human or murine bone marrow (BM)-derived PMNs were added to the upper chamber (1×10^6^ per well) and induced to chemotax by the addition of an fMLF gradient to the lower chamber (200nM in 500µL HBSS+, 37°C). The number of migrated PMNs in the lower chamber following 30 minutes (for human PMNs) and 2hr (for murine PMNs) migration was quantified by counting of 7-10 randomly selected fields of view using an inverted phase-contrast microscope (Leica Microsystems; Wetzlar, Germany).

### Adhesion Assay

BM-derived murine or human peripheral blood PMNs were labeled with CellTracker™ Green or Orange CMFDA (per manufacturer instructions) and added (3×10^5^ cells) to ECs (grown to confluence in 96-well plates) in the presence of fMLF (200nM for human and 500nM for murine PMNs) stimulation with and without the relevant inhibitors. Following 30min incubation (37°C), EC monolayers were washed x3 times and the remaining adherent PMNs were quantified by counting of 7-10 randomly selected fields of view. For transwell and adhesion assays counts were performed equally by 2 investigators in a blinded fashion.

### Viability Assays

For* ex vivo* assessment of WT and MPO KO PMN viability, freshly isolated BM-PMNs (5×10^5^ cells per condition) were incubated in RPMI 1640 in low adhesion 24-well plates (CoStar Group, Washington DC) with/without fMLF pre-stimulation (500nM, 20 min). After 24 and 48h, PMNs were stained with Annexin V and Propidium Iodide and analyzed by flow cytometry. For *in vivo* assessment, BM-derived WT and MPO KO PMNs were stained with CellTracker™ Green and Orange CMFDA, respectively (yielding green and red PMNs) and injected intravenously into WT recipient mice. Blood samples were collected at 1 and 4hrs following PMN transfer and apoptosis of endogenous, WT and MPO KO PMNs were analyzed using Annexin V staining and flow cytometry. Endogenous and transferred PMNs were identified as CD45^+^/CD11b^+^/Ly6G^+^ and red/green fluorescence negative or positive cells.

### Differential Leukocyte Counts

Fresh blood samples were obtained from WT and MPO KO mice *via* tail bleed. Following red blood cell lysis using ACK buffer, leukocytes were spun onto slides (Cytospin 4 cytocentrifuge, Thermo Scientific; Waltham, MA), fixed and stained using a Diff-Quick™ Stain Set (Siemens; Munich, Germany). All cell counts were performed in a blinded fashion and included at 20 randomly selected fields of view per condition per independent experiment. Data are shown as percent of total counted cells.

### Immunoblotting

Immunoblotting was performed as previously described (24). Briefly, BM-derived PMNs were lysed in Laemmli buffer. Proteins (10-30μg) were separated by SDS-PAGE gels (10-15%), transferred to nitrocellulose membrane and probed with relevant primary (overnight at 4°C) followed by HRP-conjugated secondary Abs (1h, RT). Peroxidase activity was detected using Immunobilon Crescendo Western HRP Substrate (Millipore).

### Statistics

Statistical significance was assessed by two-tailed, unpaired Student t-test or by one-way ANOVA with Newman-Keuls Multiple Comparison Test using Graphpad Prism (V4.0). Statistical significance was set at p<0.05. For all experiments the data shown as ± SEM.

## Results

### PMN Tissue Localization Is Enhanced in the Absence of Functional MPO

**Figure 1:**
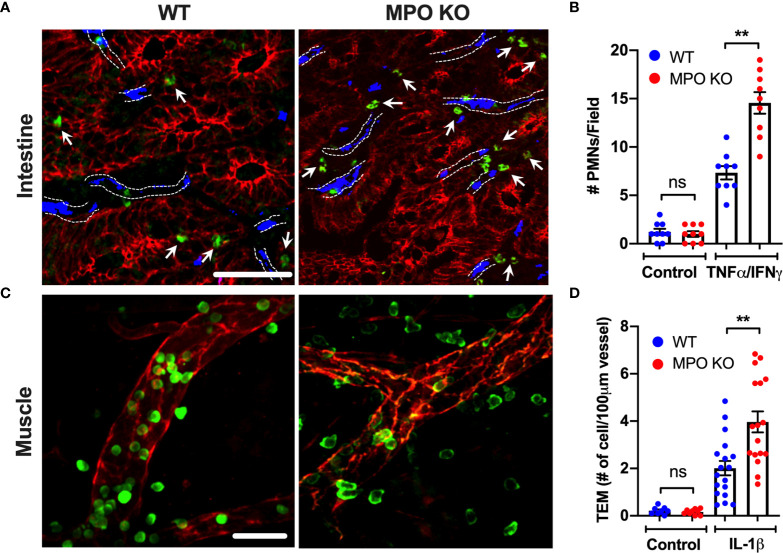
PMN tissue localization is enhanced in the absence of functional MPO. **(A, B)** Inflammation of the intestinal mucosa was induced by intraperitoneal administration of TNFα and IFNγ (500ng, each 24hr). Immunofluorescence analyses were performed on OCT-frozen 10µm-sections. PMNs, intestinal epithelial cells and blood vessel were visualized by S100A8 (green), E-Cadherin (red) and PECAM (CD31, blue) staining respectively. **(A)** Representative images and **(B)** quantification show increased number of tissue-infiltrating PMNs in MPO KO mice. Blood vessel locations are highlighted by dotted lines. White arrow point to tissue localized PMNs. **(C, D)** Inflammation of the cremaster muscle was induced by intrascrotal administration of IL-1β (50ng, 4hr). PMNs and ECs were visualized in a whole mount muscle preparation by respective staining with S100A8 (green) and CD31 (red). Consistently, PMN numbers in tissue were significantly elevated in MPO KO animals. N=3 independent experiments with 3 mice per condition. **p < 0.01. ns, not significant.

Following PMN activation and degranulation, MPO can associate with the cell membrane, where it can bind CD11b/CD18 (Mac1) integrin (12). Given the important role of CD11b in PMN trafficking and the potential of MPO binding to interfere with its function, recruitment of WT and MPO KO PMNs was compared in intestinal and skeletal muscle inflammation. Intraperitoneal administration of TNFα and IFNγ (500ng each, ip.) and intrascrotal injection of IL-1β (50ng) were used to stimulate inflammation in the intestinal mucosa and the cremaster muscle, respectively (23, 25). While PMNs are absent in unstimulated (control) WT and MPO KO mouse tissue, inflammatory stimulation resulted in ~2-fold increase in the number of tissue PMNs in both the intestinal lamina propria (**Figures 1A, B**) and the muscle tissue (**Figures 1C, D**) in MPO KO as compared to WT mice. These data suggest that the presence of MPO attenuates PMN tissue accumulation, whereas its removal relieves the inhibition.

### MPO Expression Does Not Impact PMN Viability

**Figure 2:**
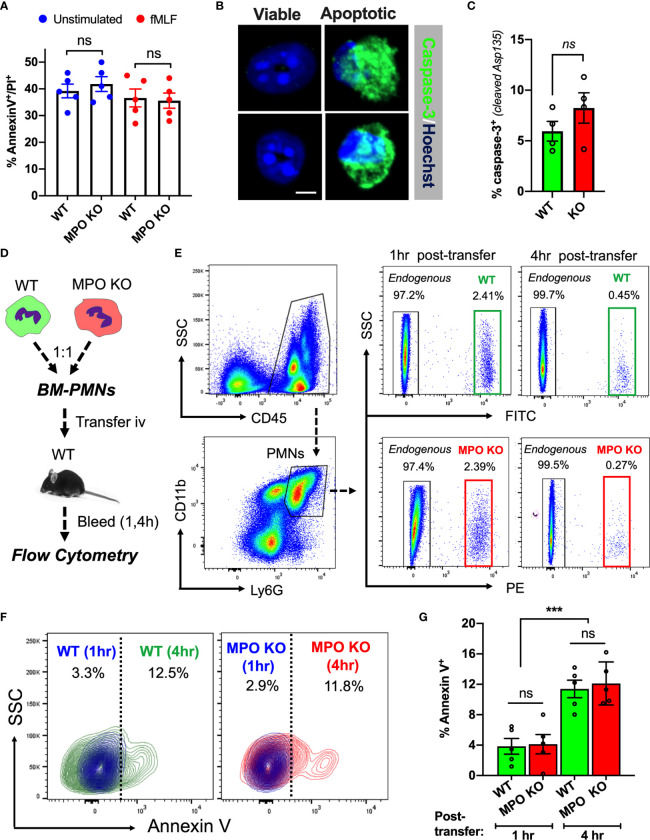
MPO expression does not impact PMN viability. **(A)** PMN viability was assessed in *ex vivo* cultured BM-derived PMNs with/without fMLF-stimulation using Annexin V/PI staining and flow cytometry. Data shown as percent Annexin V/PI positive cells at 24hr in cultures. No significant differences between MPO KO and WT mice were observed. N=5 independent experiments. **(B, C)** Representative images and quantification of cleaved caspase-3 in fMLF-stimulated BM-derived WT and MPO-KO PMNs. No significant differences between MPO KO and WT mice were observed. N=4 independent experiments. The bar is 5µm. **(D–G)** PMN viability was examined *in vivo* in adoptively transferred PMNs. **(D)** Schematic depicting experimental timeline. Freshly isolated murine WT and MPO KO BM-PMNs were respectively labeled with green and red fluorescence (CellTracker) and injected intravenously at a 1:1 ratio into WT recipients that were pre-stimulated with IL-1β (50ng, 1hr, ip., to induce systemic inflammation). Annexin V staining and flow cytometry was used to gauge viability of transferred and endogenous PMNs at 1 and 4hr. **(E–G)** Representative flow diagrams of the gating strategy to identify transferred and apoptotic WT and MPO KO PMNs and quantification of apoptotic (AnnexinV-positive) PMNs in the circulation. No significant difference in viability between MPO KO and WT PMNs. N=5 mice per condition. ns, not significant. ***p < 0.001.

Increased tissue accumulation can be driven by delayed cell apoptosis/increased survival or enhanced migratory ability. Indeed, exogenous MPO has been suggested to delay PMN apoptosis *via* activation of CD11b-dependent signaling (26). As such, we asked whether PMN viability was impacted by the lack of MPO expression. *Ex vivo* cultures of WT and MPO KO bone marrow-derived PMNs (BM-PMNs) with or without fMLF stimulation (500nM) showed no significant difference at 24hr (**Figure 2A**) to 48hr (at 48hr ~80 percent of PMNs were found to become apoptotic). Further assessment of cell apoptosis using cleaved Caspase-3 (Asp-175) by immunofluorescence similarly revealed no significant differences in fMLF-stimulated WT and MPO-KO PMNs (**Figures 2B, C**).

To test whether MPO impacts the lifespan of circulating PMNs under physiological shear conditions, WT and MPO KO BM-PMNs were stained with green and red fluorescence respectively and injected intravenously into WT-recipients (2x10^6^ PMNs, 1:1 ratio), which were conditioned by IL-1β injection (50ng, ip. 1hr prior to PMN transfer) to induce inflammation. Apoptosis of transferred WT and MPO KO PMNs in inflamed circulation was examined by flow cytometry on whole blood sample 1- and 4- hours post-transfer as indicated in the schematic (**Figure 2D**). A substantial population of transferred WT (green) and KO (red) PMNs was detected at 1hr, however at 4hr, the majority (>80%) of transferred PMNs were similarly cleared from the circulation in WT and MPO KO mice (gating is shown in **Figure 2E**). Importantly, while the number of transferred Annexin V positive PMNs significantly increased over time, no significant difference was found between WT and MPO KO PMNs (**Figures 2F, G**). Although these data do not rule out the possibility that MPO may impact the lifespan of PMN in the tissue, they indicate that increased numbers of MPO KO PMNs in inflamed tissue could be driven by enhanced migratory ability of MPO KO PMNs and not due to increased survival in the circulation.

### MPO KO PMNs Exhibit Enhanced Migratory Efficiency

**Figure 3:**
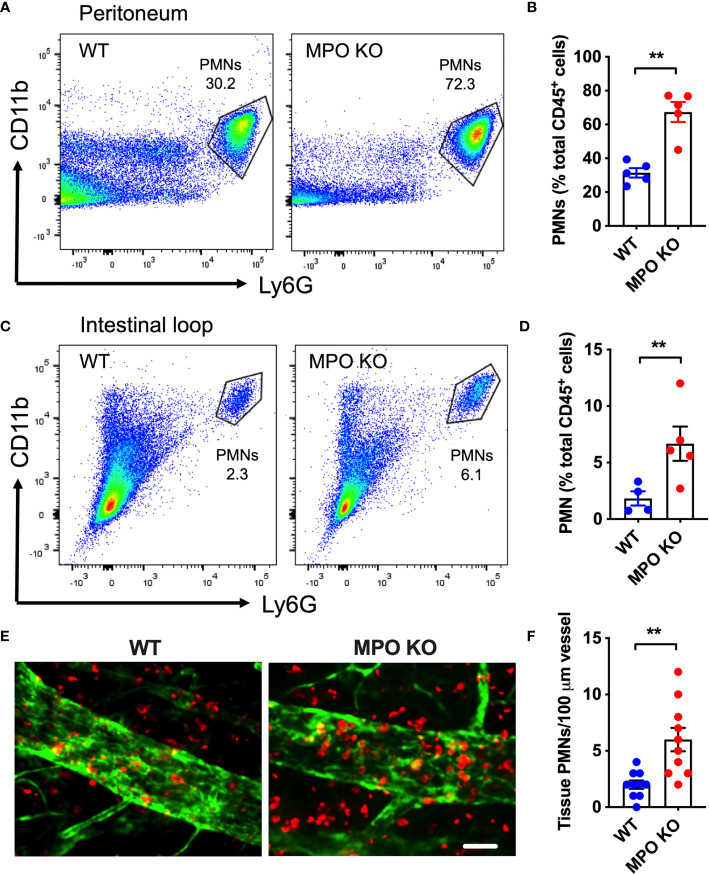
MPO KO PMNs exhibit elevated migratory efficiency. Two *in vivo* PMN models were used to assess the migratory efficiency of WT and MPO KO PMNs. **(A, B)** Zymosan-induced peritonitis model was used. **(A)** Representative flow diagrams of lavaged PMNs that have migrated into the peritoneal cavity and **(B)** Quantification demonstrate enhanced PMN migration in the absence of MPO. **(C–F)** Intestinal loop model, where PMN migration into the lumen of ligated intestinal segments is induced by intraluminal CXCL1 administration was used to assess the impact of MPO on PMN migration. **(C)** Representative flow diagrams of lavaged luminal PMNs and **(D)** Quantification revealed consistently increased PMN tissue infiltration in MPO knockout mice. **(E)** Representative fluorescence images of intestinal whole mount tissue following lavage, stained for PMNs (red, S100A8) and ECs (green, CD31) and **(F)** Quantification similarly depict increased PMN infiltration in MPO KO mice. The bar is 50µm. N=5-7 mice per condition. **p < 0.01.

To determine whether MPO expression impacts PMN migration, zymosan-induced peritonitis and ileal loop PMN migration assays were performed. Both assays allow for quantification of acute PMN mobilization from the blood vessels into the tissue by flow cytometry and as such directly test the PMN migratory efficiency. Zymosan treatment (ip. 2µg/ml, 4h) as well as CXCL1 administration (a potent PMN chemoattractant, 1µg/ml, 2h) into the lumen of ligated ileal loops induced a rapid PMN mobilization into the peritoneal cavity (**Figures 3A, B**) and the intestinal lumen (**Figures 3C, D**), respectively. Importantly, in both models, PMN migration was significantly enhanced in the absence of functional MPO. Elevated tissue infiltration by MPO KO PMNs was further evident from whole-mount immunofluorescence staining and confocal microscopy imaging of the ileal loop tissue (**Figures 3E, F**).

### MPO KO PMNs Exhibit Enhanced Adhesion to Inflamed Vascular Endothelial Cells

**Figure 4:**
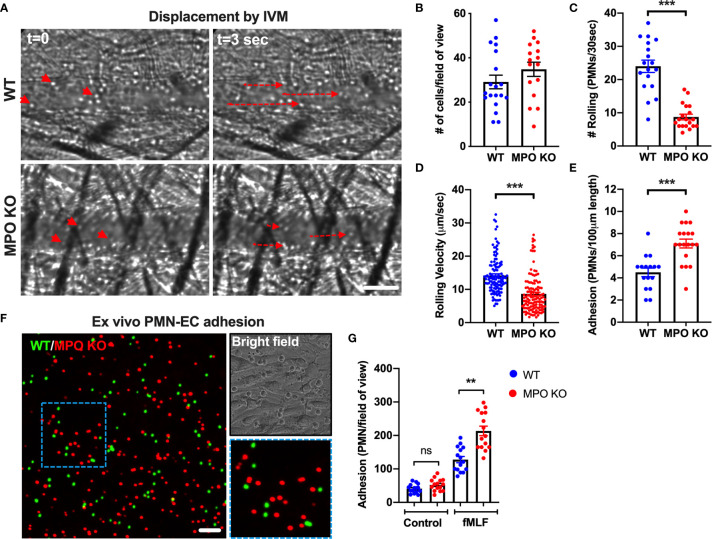
MPO KO PMNs exhibit enhanced adhesion to inflamed vascular endothelial cells. **(A–E)** To assess the role of MPO in PMN-EC interactions intravital imaging (IVM) of the cremaster muscle was performed on WT and MPO KO mice. Inflammation of the cremaster muscle was induced by intrascrotal administration of IL-1β (50ng, 4hr). **(A)** Representative time-lapse images (based on a real-time acquisition) show decreased displacement of rolling PMNs in MPO KO animals. The bar is 50µm. **(B)** Analyses of total cell fluxes **(C)** Number of rolling cells per 30 seconds **(D)** Rolling velocities of individual PMNs and **(E)** PMN adhesion reveal a significant reduction in rolling and increases in adhesive interactions in MPO KO mice. N=5 mice with 5-6 fields of view per condition. **(F–G)** *Ex vivo* adhesion assay of BM-PMNs and murine ECs was performed. WT and MPO KO BM-PMNs were respectively stained with green and red fluorescence (CellTracker) and allowed to adhere to TNFα-stimulated (50ng, 24hr) Bend3 ECs in the presence of fMLF stimulation (500nM). **(F)** Representative fluorescent images and **(G)** Quantification reveal significant increases in MPO KO adhesion compared to WT. The bar is 50µm. N=3-5 independent experiments per condition. **p < 0.01, ***p < 0.001.ns, not significant.

Rolling and firm adhesion of circulating PMNs are prerequisite steps for the crossing of the endothelial barrier and infiltration of the surrounding tissue. Thus, we performed high-speed intravital microscopy (IVM) using bright field illumination to assess in real-time the impact of MPO on PMN interactions with inflamed vasculature *in vivo* (**Figure 4A**). Localized inflammation of the cremaster muscle was induced by intrascrotal administration of IL-1β (50ng, 4hr). Intravital imaging revealed that the total number of circulating PMNs, was not different in WT vs MPO KO mice (**Figure 4B**). However, the number of rolling PMNs and the rolling velocity were significantly reduced in MPO KO mice **(** **Figures 4C, D**). Increased displacement of representative rolling WT compared to MPO KO PMNs are shown by time-lapse images, indicating higher rolling velocity (**Figure 4A** and **Supplemental Movies 1** and **2**). Consistent with reduced rolling fluxes, a significantly higher number of adhered PMNs (~2-fold) was observed in MPO KO as compared to WT mice (**Figure 4E**). Increased adhesion of MPO KO PMNs to vascular endothelium support the observed increases in tissue PMNs, confirming a role of MPO in PMN migration. Of note, blood differential analyses by Wright-Giemsa staining revealed no difference in the number of circulating lymphocytes and monocytes as well as in the number of circulating segmented (mature) and banded (immature) PMNs (**Supplemental Figure 1**). This indicates that production and release of PMNs into circulation was not impacted by the lack of MPO.

Supporting *in vivo* observations of circulating PMNs, MPO KO BM-PMNs exhibited increased adhesion to ECs in a complementary *ex vivo* static adhesion assay. Incubation of mixed WT and MPO KO BM-PMNs (respectively labeled with green and red fluorescence, at 1:1 ratio) with murine Bend3 ECs in the presence of fMLF stimulation (to enhance CD11b activation and PMN adhesion) revealed higher number of adhered MPO KO compared to WT PMNs (**Figures 4F, G**). The relatively low baseline PMN adhesion without fMLF-stimulation was also not different between WT and MPO KO PMNs.

### CD11b Surface Expression Is Not Altered by the Absence of MPO

**Figure 5:**
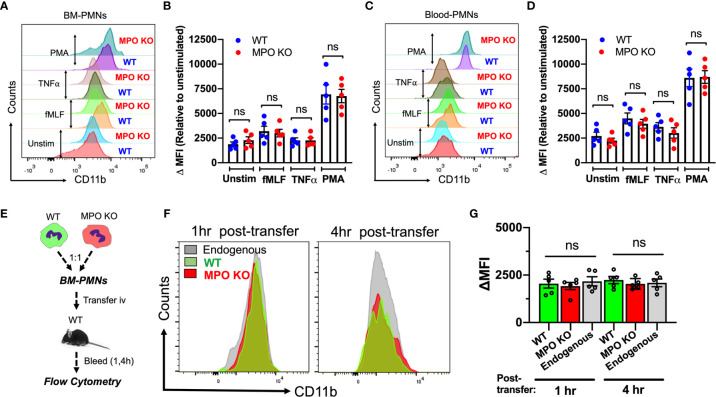
CD11b surface expression is not altered by the genetic deletion of MPO. **(A–D)** CD11b expression was assessed by flow cytometry in BM-derived and circulating blood PMNs with/without *ex vivo* inflammatory stimulation. **(A, B)** Representative flow diagram and quantification of CD11b expression in BM-PMNs. **(C, D)** Representative flow diagram and quantification of CD11b expression in circulating PMNs. N=5 independent repeats per condition. **(E–G)** CD11b expression was assessed in fluorescently labeled WT (green) and MPO KO (red) PMNs at 1 and 4hr following adoptive intravenous transfer into IL-1β-stimulated (50ng, 1hr) WT recipient mice. **(E)** Schematic depicting experimental time line. **(F, G)** Representative flow diagrams and quantification reveal no significant differences in CD11b expression. N=5 mice per condition. ^ns^ not significant.

CD11b is the most abundant and prominent adhesive receptor in PMNs, mediating both PMN adhesion and migration in inflammation (20, 27). As such, elevated levels of surface CD11b could account for the elevated adhesion and migration of MPO KO PMNs. To test this, we compared the CD11b surface expression (non-permeabilized conditions) on BM-derived and peripheral blood WT and MPO KO PMNs under resting and stimulated conditions. Interestingly, although PMN stimulation as expected elevated CD11b levels, no significant differences in expression patterns were noted (**Figures 5A–D**). To further examine this under physiological shear conditions, CD11b expression was tested in WT and MPO KO PMNs following their adoptive transfer into the circulation of inflamed recipient mice (as detailed in **Figure 2** and schematic **Figure 5E**). In this model too, under physiological shear conditions and inflammation, no significant differences in CD11b expression in transferred WT and MPO KO PMNs were noted (**Figures 5F, G**).

To further explore potential mechanisms of MPO-dependent increases in adhesion and migration of MPO KO PMNs, we compared the expression of CD11a, another important integrin receptor involved in PMN adhesive interactions as well as key PMN chemokine receptors CXCR2 and CXCR4 in WT and MPO KO PMNs. We found no significant differences in unstimulated or fMLF-stimulated PMNs (**Supplemental Figures 2A–C**). Similarly, immunoblotting analyses reveled no differences in extracellular signal-regulated kinase (ERK) activation (phosphorylation) between WT and MPO KO PMNs (**Supplemental Figure 2D**). ERK plays an important role in PMN chemotaxis (28) and can be activated downstream of CD11b (29).

### Exogenous MPO Attenuates PMN Adhesion to Inflamed Endothelium

**Figure 6:**
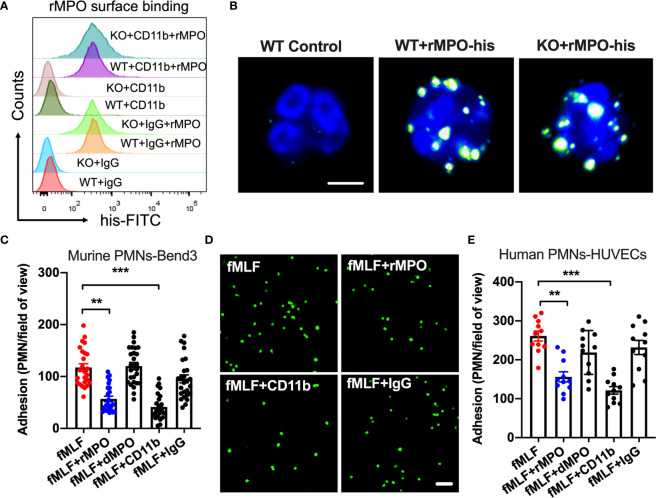
Free MPO attenuates PMN adhesion to inflamed endothelium. **(A)** To assess MPO binding to the PMN surface and whether it is CD11b dependent, BM-PMNs were incubated with His-tagged recombinant MPO (rMPO, 10µg/ml 30min) in the presence of fMLF (500nM, to induce CD11b expression and activation) and with/without the addition of control IgG and anti-CD11b inhibitory Abs. MPO binding analyses were examined by flow cytometry on non-permeabilized PMNs and detection of His-tag. Robust MPO-His binding to PMNs was seen, however this was independent of CD11b expression. Representative of N=5 independent repeats. **(B)** The binding of His-tagged recombinant MPO to WT and MPO KO BM-PMNs was further confirmed by immunofluorescence. Surface-bound MPO was detected by anti-His antibody staining. The bar is 5µm. **(C, D)** To establish whether MPO impairs PMN adhesion, BM-PMNs were fluorescently stained (CellTracker Green) and incubated with TNFα-stimulated (50ng, 24hr) Bend3 ECs in the presence of fMLF stimulation (500nM) and with/without the addition of intact or control denatured (by boiling) rMPO. **(C)** Quantification and **(D)** Representative images reveal decreased adhesion in the presence of rMPO and CD11b inhibitory Ab but not the IgG control or denatured rMPO protein. The bar is 50µm. N=5 independent repeats in duplicates with at 3-5 randomly selected fields analyzed per condition. **(E)** Similar adhesion assays were performed using freshly isolated human PMNs and HUVECs, revealing consistent inhibition of PMN adhesion by rMPO. N=3 independent repeats in triplicates. **p < 0.01, ***p < 0.001.

Since we found no difference in integrin/chemokine expression and signaling, we next tested the idea that released/exogenous MPO may competitively suppress PMN adhesion and migration by binding to the PMN surface. We first used flow cytometry and immunofluorescence/confocal microscopy to confirm that exogenous His-tagged recombinant MPO (rMPO) indeed robustly binds to the PMN surface (**Figures 6A, B**). Although rMPO has been shown to interact with CD11b and promote CD11b-dependent signaling (12) surprisingly, inhibitory Ab to CD11b did not significantly attenuate rMPO binding, suggesting that the MPO binding is not specific to CD11b.

We next examined the direct impact of MPO on PMN adhesion to inflamed ECs. To this purpose, WT BM-PMNs (5x10^5^ cells/condition) were incubated with TNFα-stimulated murine endothelial cells (ECs, Bend3) in the presence or absence of murine rMPO. PMN adhesion was significantly reduced (~2-fold) in the presence of rMPO (**Figure 6C** and representative images, **Figure 6D**). As expected, in control experiments, Ab-mediated inhibition of CD11b, which is an important contributor to PMN-EC adhesion, but not the addition of control IgG suppressed PMN adhesion to endothelial cells. Similar observations were made with freshly isolated human PMNs and ECs. The addition of human rMPO or an inhibitory anti-CD11b Ab but not denatured protein or IgG control Ab significantly inhibited PMN adhesion to HUVECs (**Figure 6E**). These observations support that idea that MPO binding sterically interferes with PMN adhesion to reduce PMN migration.

### MPO Impairs *Ex Vivo* and *In Vivo* PMN Migration

**Figure 7:**
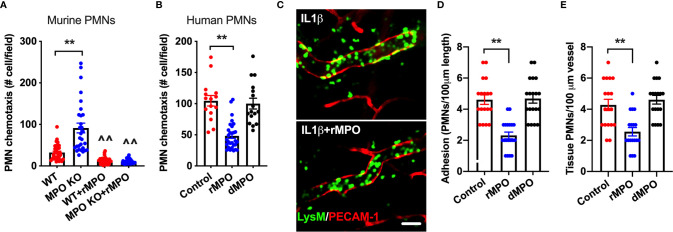
MPO impairs *ex vivo* and *in vivo* PMN migration. **(A, B)** To test whether MPO-mediated impairment of PMN adhesion resulted in inhibition of PMN migration, *ex vivo* chemotaxis assays using transwells and murine BM-derived and human blood PMNs were performed. For all conditions PMNs chemotaxis was induced by the addition of fMLF to the bottom chamber. **(A)** Chemotaxis of MPO KO murine PMNs was significantly elevated compared to WT cells and was significantly suppressed with the addition of rMPO (20µg/ml). **(B)** Similarly, migration of human PMNs was inhibited by the addition of rMPO. N=5 independent repeats in triplicates. **(C, E)** IVM on the cremaster muscle in LysM-eGFP reporter mice (green PMNs) was performed to assess the impact of rMPO administration on PMN adhesion and TEM. Inflammation of the cremaster muscle was induced by intrascrotal administration of IL-1β (50ng, 4hr). rMPO (30µg in 100ml of sterile PBS) was administered retro-orbitally immediately following IL-1β stimulation. A significant reduction in **(D)** PMN adhesion and **(E)** TEM (extravasated PMNs in the tissue) was seen. The bar is 50µm. N=5 mice per condition with 3-5 randomly selected fields. **p < 0.01, ^^ significantly different from non-treated controls, p < 0.01.

Given the suppressive action of MPO on PMN adhesion, we postulated that this will lead to inhibition of PMN migration. To test this, chemotaxis assays [using a transwell setup (23)] were performed. In these experiments, murine WT and MPO KO PMNs or human PMNs were introduced to the upper chamber and were induced to chemotax across permeable supports by the addition of an fMLF gradient to the bottom chamber. Consistent with increased adhesion, chemotaxis of murine MPO KO PMNs was significantly elevated compared to WT PMNs (**Figure 7A**). Furthermore, supporting the idea that MPO binding to the PMN surface impairs PMN interactions with substratum, the addition of rMPO, but not a denatured protein, to either murine or human PMNs significantly reduced PMN chemotaxis (**Figures 7A, B**).

Finally, as with *ex vivo* observations, the suppressive impact of MPO on PMN adhesion and TEM was confirmed *in vivo*, using cremaster muscle IVM. In these experiments, LysM-eGFP reporter mice (green PMNs and macrophages) were used and the vasculature was outlined by PECAM-1 fluorescence staining, allowing for clear separation of intravascular vs extravasated PMNs by fluorescence. Likewise, tissue macrophages are easily distinguishable from extravasated PMNs based on their size (significantly larger cells) and ramified morphology. Inflammation of the cremaster muscle was induced by IL-1β as in **Figure 4**. Intact or control denatured murine rMPO (30µg MPO, iv. 4hr) was administrated immediately following IL-1β stimulation. Consistent with the hypothesized role of MPO in impairment of PMN trafficking, significant reduction in the number of adherent and extravasated (tissue) PMN was observed with the addition of intact but not denatured rMPO (representative images **Figures 7C**–**E** and **Supplemental movies 3** and **4**). These experiments confirm the inhibitory role of MPO in the regulation of PMN migration in inflammation.

## Discussion

MPO is the most abundantly expressed protein in PMNs and its primary physiologic function is the killing of invading microorganisms. Together with H_2_O_2_, MPO forms a powerful antimicrobial system, that when released into the phagosomes, help kill the ingested bacteria *via* generation of hypochloric acid (HOCl) and other reactive oxidative radicals. While the anti-microbial function of MPO is mostly intracellular [with the exception of NETosis where MPO assists in catching and eliminating pathogens outside the cells (30, 31)], the pro-and-anti-inflammatory effects of MPO take place once it is released outside the cell. Indeed, in addition to the antimicrobial function of MPO other important roles of MPO in tissue homeostasis and cellular function have been identified.

In the current work, we demonstrate that MPO can directly bind to the PMN surface and attenuate PMN adhesion and migration in inflammation. Using *in vivo* models of inflammation and *ex vivo* murine and human PMN co-cultures with vascular ECs, we show that in the absence of functional MPO, PMN adhesion and migration is significantly enhanced. In contrast, in the presence of soluble MPO, PMN adhesion and migration were substantially suppressed. Thus, our findings suggest that MPO may play a protective role in inflammation by limiting PMN tissue accumulation.

There are plenty of data showing that under inflammatory conditions, MPO is spilled into the circulation, as elevated free MPO plasma levels were detected in many pathological conditions including sepsis and acute coronary syndromes (12).

Once released by PMNs, free MPO can bind vascular ECs and contribute to endothelial dysfunction by limiting NO bioavailability (32) or *via* HOCl-mediated modification of L-arginine (33). Interestingly, *ex vivo*, human PMNs were able to adhere to immobilized recombinant MPO (14) suggesting that MPO bound to vascular EC, could exhibit adhesive properties augmenting PMN-EC interactions. However, *in vivo*, MPO bound to ECs is rapidly transcytosed across the endothelium to modulate extracellular matrix proteins in inflammation (34). In our setting, administration of free MPO into inflamed microcirculation reduced rather than augmented PMN adhesion, supporting an inhibitory role of MPO in PMN recruitment. PMN adhesion to the vascular endothelium is a prerequisite and necessary step for the subsequent PMN TEM. Adhesive events both guide PMNs to the specific location/portals where TEM can take place (35) as well as transduce essential signaling events in EC (via engagement of EC surface ligands such as ICAM-1 and PECAM-1) to accommodate PMN crossing (36, 37). As such, it is not surprising that impaired PMN adhesion in the presence of soluble MPO led to reduced tissue infiltration by PMNs. This novel regulatory role of MPO in PMN trafficking is of potential therapeutic relevance, given the interest in neutrophil trafficking as potential therapy approaches, and the ongoing debate on the pro-and anti-inflammatory functions of PMNs in inflammation.

Indeed, although MPO presence in tissues is mainly associated with inflammation and injury, protective functions of MPO were also identified. For example, MPO activity and the presence of its oxidative products have been implicated in glomerular injury (38, 39), atherosclerosis (40), lung damage in CF (41) and carcinogenesis (42). Free MPO or MPO released in extracellular vesicles has been shown to bind epithelial cells and impair intestinal wound healing (9). MPO internalized by alveolar and bronchial epithelial cells led to DNA strand breakage, however it also suppressed interleukin-8 production, potentially limiting PMN recruitment and retention in tissue (43). Finally, MPO has been reported to protect against experimental autoimmune encephalomyelitis, an animal model for multiple sclerosis, with MPO‐knockout mice being more susceptible to these disease (44). Similarly, an unexpected protective role for MPO was found in murine atherosclerosis, where in the absence of MPO larger aortic legions and inflammation was observed (45).

Free MPO by binding to CD11b/CD18 has also been shown to induce neutrophil activation in an autocrine fashion promoting MAPK and NFκB signaling, ROS production and degranulation (12). Furthermore, MPO KO PMNs were suggested to better phagocyte zymosan particles due to slightly elevated levels of CD11b and the resulting induction of FAK/ERK signaling (13). However, by doing careful examination of CD11b expression, which included BM-PMNs, circulating blood PMNs with and without stimulation, as well as following adoptive competitive transfer of WT and MPO KO PMNs into inflamed recipient circulation we found no significant differences in CD11b expression. Consistently, although we confirmed that free MPO robustly binds to the surface of both murine and human PMNs, blockade of CD11b by inhibitory Ab did not reduce the MPO binding. These findings do not exclude the likelihood of MPO engagement with CD11b to induce signaling in PMNs, however also suggests that MPO binds other PMN surface ligands. There is also a possibility that in addition/alternatively to facilitating intracellular signaling to impact PMN adhesive interactions, MPO non-specifically coats the PMN surface to sterically hinder PMN binding to substratum and as such impeding PMN migratory ability. As such, our work reveals a new and important regulatory function of MPO in PMN migration, serving to “brake” excessive PMN accumulation in tissues. Our findings further provide basis for future studies of mechanisms and potential binding targets of MPO at the PMN surface.

## Data Availability Statement

The original contributions presented in the study are included in the article/**Supplementary Material**. Further inquiries can be directed to the corresponding author.

## Ethics Statement

The studies involving human participants were reviewed and approved by Northwestern University Institutional Review Board. The patients/participants provided their written informed consent to participate in this study. The animal study was reviewed and approved by NORTHWESTERN UNIVERSITY Institutional Animal Care and Use Committee.

## Author Contributions

JR and RS conceived and designed experiments. JR, TB, CG-E, JU, XR, DP and RS conducted experiments and performed data analyses. JR and RS wrote the manuscript. TB, CG-E, JU, XR and DP edited the manuscript. All authors contributed to the article and approved the submitted version.

## Funding

This work was supported by grants from the American Cancer Society Research Scholar Award (RSG-17-235-01), Crohn’s & Colitis Foundation Senior Research Award (624450) and NIH/NIDDK R01 DK124199 award to RS, the Department of Defense CDMRP’s Horizon Award (CA191071) to TB and Northwestern University Undergraduate Research Grant to JR. Publication costs of this research article were supported by the Sidney and Bess Eisenberg Memorial Fund.

## Conflict of Interest

The authors declare that the research was conducted in the absence of any commercial or financial relationships that could be construed as a potential conflict of interest.
