# Supplementary figures and images for: Released Myeloperoxidase Attenuates Neutrophil Migration and Accumulation in Inflamed Tissue
Source: Front Immunol. 2021 Apr 20;12:654259. doi: 10.3389/fimmu.2021.654259 (PMC8093447; doi:10.3389/fimmu.2021.654259)

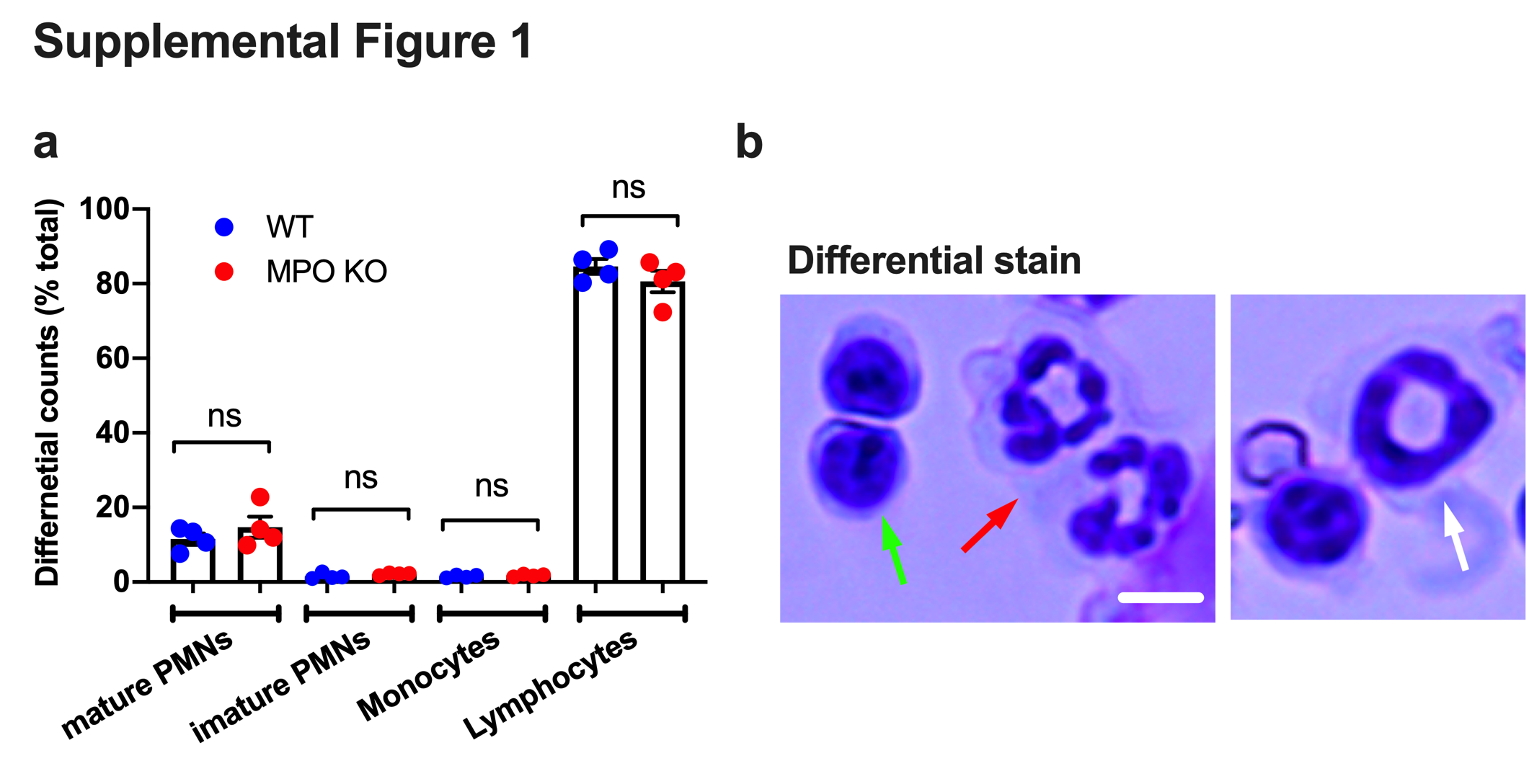

Supplement: Supplementary file 1 [file Image_1.tiff]

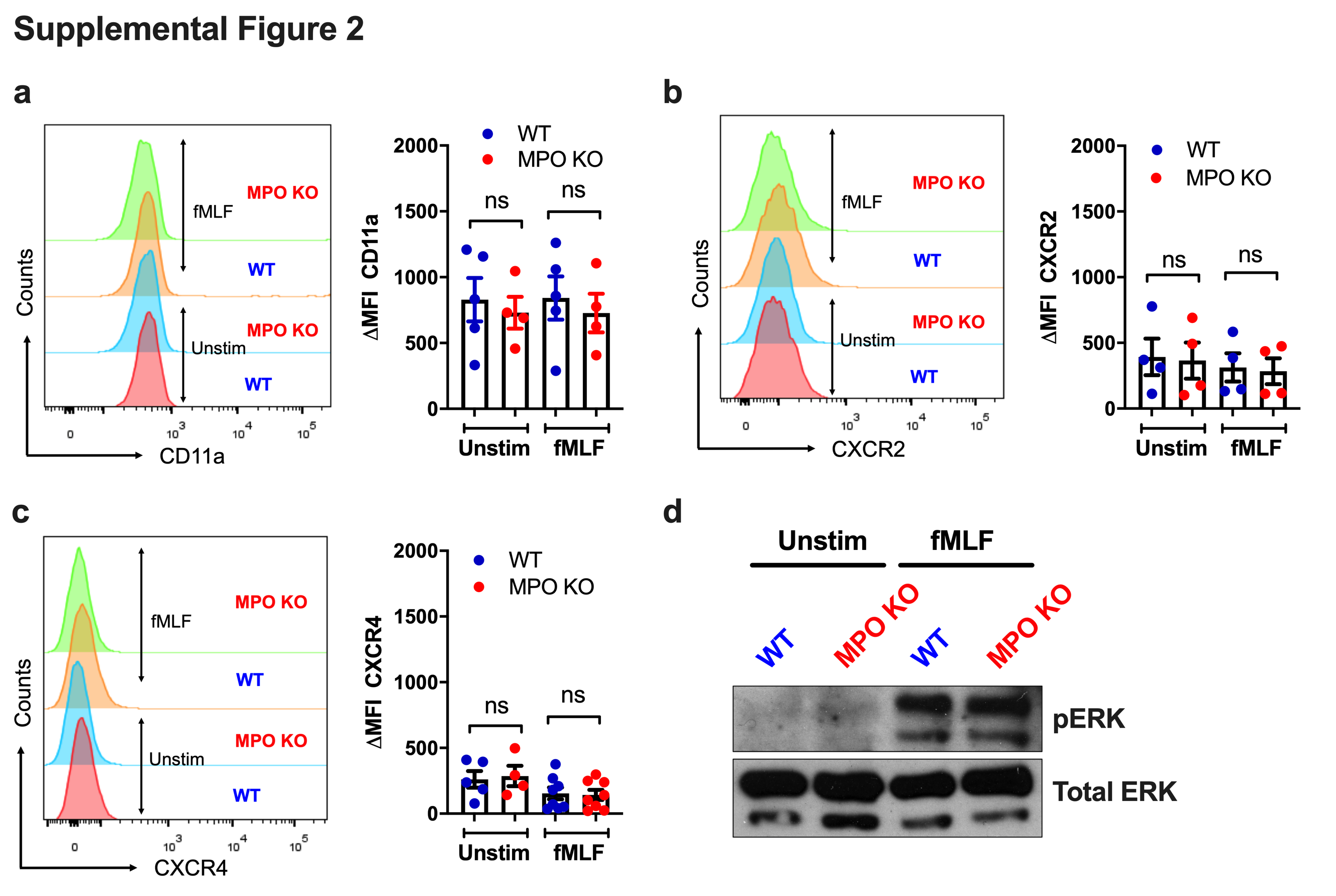

Supplement: Supplementary file 2 [file Image_2.tiff]
