# Supplementary material for: Released Myeloperoxidase Attenuates Neutrophil Migration and Accumulation in Inflamed Tissue
Source: Front Immunol. 2021 Apr 20;12:654259. doi: 10.3389/fimmu.2021.654259 (PMC8093447; doi:10.3389/fimmu.2021.654259)
Supplement: Supplementary file 3 [file Table_1.docx]

**Supplemental Figure Legends**

**Supplemental Figure 1: MPO does not impact circulating leukocyte cell counts.** Blood samples were obtained from WT and MPO KO mice. Following red blood cell lysis, circulating leukocytes were spun onto slides and stained using Wright-Giemsa differential staining. **(a)** Respective numbers of lymphocytes monocytes and mature vs immature PMNs were quantified from randomly acquired high-resolution images. No significant difference in circulating leukocyte cell counts was found between WT and MPO KO mice. **(b)** Representative images of lymphocytes (green arrow), segmented/mature PMNs (red arrow), and banded/immature PMNs (white arrow). The bar is 5μm. N=4 independent experiments. ^ns^ not-significant.

**Supplemental Figure 2: MPO deficiency does not affect CD11a, CXCR2, CXCR4 or ERK expression. (a-c)** Flow cytometry was used to analyze the surface expression of β2-integrin, CD11a, and key PMN chemokines CXCR2, and CXCR4 on BM-derived WT and MPO KO PMNs with/without stimulation. Representative flow diagrams and MFI quantification revealed no significant difference in **(a)** CD11a, **(b)** CXCR2 and **(c)** CXCR4 expression in WT compared to MPO KO mice. **(d)** Immunoblotting of unstimulated and fMLF-stimulated BM-derived PMNs demonstrates no difference in the levels of phospho- (Thr202/Tyr204) or total ERK 1/2 expression between WT and MPO KO mice. N=4 independent experiments. ^ns^ not-significant.

**Supplemental Movies**

**Supplemental movie 1:** IVM was performed on IL-1β (50ng, 4hr) stimulated cremaster muscle in WT mice. Adhered and rolling leukocytes were visualized using bright field illumination. The number of adherent leukocytes in WT mice was lower compared to MPO KO mice, whereas the number of rolling leukocytes and the rolling velocities were significantly elevated.

**Supplemental movie 2:** IVM was performed on IL-1β (50ng, 4hr) stimulated cremaster muscle in MPO KO mice. Adhered and rolling leukocytes were visualized using bright field illumination. The number of adherent cells in MPO KO mice was significantly increased and velocities of rolling leukocytes was significantly reduced compared to WT mice.

**Supplemental movie 3:**

IVM was performed on IL-1β (50ng, 4hr) stimulated cremaster muscle of LysM-eGFP reporter mice (green PMNs and macrophages). **Denatured** (control) murine rMPO (30μg MPO) was administered via retroorbital injection immediately following the induction of inflammation. Vasculature was outlined by PECAM-1 fluorescence staining, and intravascular rolling or adhered PMNs vs extravasated tissue PMNs were visualized in real-time.

**Supplemental movie 4:**

IVM was performed on IL-1β (50ng, 4hr) stimulated cremaster muscle of LysM-eGFP reporter mice (green PMNs and macrophages). **Intact** murine rMPO (30μg MPO) was administered via retroorbital injection immediately following the induction of inflammation. Vasculature was outlined by PECAM-1 fluorescence staining, and intravascular rolling or adhered PMNs vs extravasated tissue PMNs were visualized in real-time. A significant reduction in the number of adherent and extravasated (tissue) PMNs was observed with the addition of intact but not denatured rMPO.
